# Genome-Wide Association Study of Haploid Male Fertility in Maize (*Zea Mays L*.)

**DOI:** 10.3389/fpls.2018.00974

**Published:** 2018-07-17

**Authors:** Hailin Ma, Guoliang Li, Tobias Würschum, Yao Zhang, Debo Zheng, Xiaohong Yang, Jiansheng Li, Wenxin Liu, Jianbing Yan, Shaojiang Chen

**Affiliations:** ^1^Key Laboratory of Crop Heterosis and Utilization, Ministry of Education, Department of Crop Genetics and Breeding, National Maize Improvement Center, China Agricultural University, Beijing, China; ^2^Beijing Key Laboratory of Crop Genetic Improvement, Department of Crop Genomics and Bioinformatics, National Maize Improvement Center, China Agricultural University, Beijing, China; ^3^State Plant Breeding Institute, University of Hohenheim, Stuttgart, Germany; ^4^Maize Research Institute, Guangxi Academy of Agricultural Science, Nanning, China; ^5^National Key Laboratory of Crop Genetic Improvement, Huazhong Agricultural University, Wuhan, China

**Keywords:** maize, doubled haploids, haploid male fertility, genome-wide association study, single-nucleotide polymorphism

## Abstract

Large-scale application of the doubled haploid (DH) technology by *in vivo* haploid induction has greatly improved the efficiency of maize breeding. While the haploid induction rate and the efficiency of identifying haploid plants have greatly improved in recent years, the low efficiency of doubling of haploid plants has remained and currently presents the main limitation to maize DH line production. In this study, we aimed to assess the available genetic variation for haploid male fertility (HMF), i.e., the production of fertile pollen on haploid plants, and to investigate the underlying genetic architecture. To this end, a diversity panel of 481 maize inbred lines was crossed with “Mo17” and “Zheng58,” the F_1_ hybrids subjected to haploid induction, and resulting haploid plants assessed for male fertility in two environments. Across both genetic backgrounds, we observed a large variation of HMF ranging from zero to ~60%, with a mean of 18%, and a heritability of 0.65. HMF was higher in the “Mo17” than in the “Zheng58” background and the correlation between both genetic backgrounds was 0.68. Genome-wide association mapping identified only few putative QTL that jointly explained 22.5% of the phenotypic variance. With the exception of one association explaining 11.77% of the phenotypic variance, all other putative QTL were of minor importance. A genome-wide prediction approach further corroborated the quantitative nature of HMF in maize. Analysis of the 14 significantly associated SNPs revealed several candidate genes. Collectively, our results illustrate the large variation of HMF that can be exploited for maize DH breeding. Owing to the apparent genetic complexity of this trait, this might best be achieved by rapid recurrent phenotypic selection coupled with marker-assisted selection for individual QTL.

## Introduction

Maize (*Zea mays* L.) is one of the most important food, feed and industrial crops worldwide. With the growing demand for production, maize breeders continue to explore and improve modern breeding techniques. One of these is the doubled haploid (DH) technology, the large-scale application of which has greatly improved the efficiency of maize breeding in recent years, as it enables the rapid generation of completely homozygous lines. DH breeding consists of three main steps: induction of haploid kernels, identification of the haploid seeds or seedlings, and doubling of the haploid plants (De La Fuente et al., 2013). With the constant improvement of the efficiency of haploid induction and haploid identification, the haploid doubling efficiency has become one of the major constraints to the utilization of the DH technology in maize breeding.

Currently, haploid doubling is highly dependent on chemicals, such as colchicine or alternative chemical reagents (Barnabás et al., 1999; Kato, 2002; Pintos et al., 2007; Hantzschel and Weber, 2010). Eder and Chalyk (2002) reported that with the colchicine-induced genome doubling 49% of the treated haploid plants produced fertile pollen and 27% produced viable seeds. This approach, however, is time consuming, costly and colchicine itself is a hazardous chemical. Interestingly, genetic variation exists for fertility of haploid plants through spontaneous chromosome doubling, that may be exploited as an alternative to chemical treatments (Chase, 1952b; Geiger et al., 2006; Wu et al., 2014). For instance, Barnabás et al. (1999) reported a spontaneous doubling rate in different maize germplasm ranging from 0 to 21.4%. For comparison, the spontaneous doubling rate was found to be 10 to 40% in rapeseed (*Brassica campestris* L.) (Henry, 1999), around 30% in triticale (× *Triticosecale* Wittmack L.) (Würschum et al., 2012), and even up to 87% in some genotypes of barley (*Hordeum vulgare* L.) (Hoekstra et al., 1993). Consequently, exploiting spontaneous genome doubling for doubled haploid generation may allow to forgo the use of artificial treatments and at the same time increase the efficiency of DH production (Kleiber et al., 2012).

Notably, maize is a monoecious plant, i.e., the male and female reproductive organs are separated, with the male flowers forming on the tassel at the top of the plant. Regarding spontaneous haploid fertility, haploid female fertility occurs much more frequently than its counterpart the haploid male fertility (HMF). Previous studies showed that ears of haploid plants when pollinated with pollen from diploid inbred lines almost all carried kernels (Chalyk, 1994; Geiger et al., 2006). By contrast, the average rate of HMF is no more than 10%, but previous studies also reported much higher values in certain genotypes, ranging up to 65% (Chase, 1952a; Chalyk, 1994; Geiger et al., 2006; Liu and Zhao, 2010; Geiger and Schönleben, 2011; Kleiber et al., 2012; Wu et al., 2014). Thus, the exploitation of spontaneous fertility in maize DH production mainly depends on the availability of fertile pollen and consequently on the identification of genotypes possessing a high haploid male fertility.

Little is known to date on the genetic control underlying haploid male fertility. Wu et al. (2017) investigated four traits related to HMF based on 20 inbred lines and 31 single crosses derived from Chinese elite maize germplasm and found that HMF is controlled by two or more genes mostly showing additive gene action (Wu et al., 2017). Furthermore, Ren et al. (2017) employed a segregation distortion method in two selected haploid populations and reported three and four QTL in the “4F1/Zheng58” and “Yu87-1/Zheng58” populations, respectively. In addition, fine-mapping was performed for the key QTL, *qhmf4*, located on chromosome 6, which showed the strongest segregation distortion in both populations (Ren et al., 2017).

The aim of this study was to identify genotypes with a high HMF and to improve our understanding of the genetic architecture underlying this important trait. To this end, we employed a large diversity panel composed of 481 inbred lines that were assessed for their HMF in two genetic backgrounds and genotyped with high-density genome-wide markers for association mapping. In particular, our objectives were to (1) investigate the available diversity of haploid male fertility, (2) identify genomic regions significantly associated with restoration of male fertility in haploid lines, and (3) draw conclusions for DH breeding in maize.

## Materials and methods

### Plant germplasm and experimental design

The diversity panel used in this study consisted of 513 global diverse maize inbred lines (AM513), originating from CIMMYT, China and USA, and representing tropical, subtropical and temperate germplasm. The AM513 panel has been described in detail in previous studies (Yang et al., 2011, 2014). The haploid inducer “CAU5” (Xu et al., 2013) was bred by our laboratory. Its induction rate is stably at around 10% and its clear color marker enables the identification of haploid seeds.

All 513 maize inbred lines were planted at Nanbin Agricultral Station (N18°21′7″, E109°10′20″), Hainan, China, in 2010, in single-row plots of 2.5 m length and spaced 0.67 m apart, with 11 plants per row. During flowering time, three to five plants were selected to be crossed with “Mo17” and “Zheng58.” Hybrid F_1_ seeds were harvested and the following year were crossed with the inducer line “CAU5” at CAU Shangzhuang Breeding Station (N40°08′16″, E116°10′37″), Beijing, Nanbin Agricultral Station (N18°21′7″, E109°10′20″), Hainan, and Breeding Station of Guangxi Academy of Agricultural Sciences (N22°36′37″, E108°13′51″), Guangxi. Finally 481 of the lines from the diversity panel produced enough haploid seeds for further study. These 481 maize lines can be classified into four subgroups based on population structure: stiff stalk (SS) with 46 lines, non-stiff stalk (NSS) with 108 lines, tropical-subtropical (TST) with 206 lines, and an admixed group with 121 lines (Figure S1; Yang et al., 2011; Li et al., 2012). The two resulting panels are subsequently referred to as “Mo17” and “Zheng58” association panels. Haploid male fertility evaluation was conducted with these panels in summer 2012 and 2013 at Linze Orient Breeding Station (N39°10′56″, E100°10′3″) in Gansu, China. Completely randomized design was conducted with single seed per hole. Each plot consisted of a single row of 0.6 m in width and 7.5 m in length, in which 50 haploid F_2_ seeds from each F_1_ × “CAU5” cross were planted each year. The field management included rigorous removal of weeds and insecticide treatment. The haploid identification accuracy at seed state by color is about 90%, and during the 7–8 leaf stage, the non-haploid plants were identified and removed, then the number of haploid plants was recorded.

### Phenotyping and statistical analyses

Haploid male fertility was assessed during the pollen shedding period. Only when pollen was produced by a haploid plant that was visible to the unaided eye, then the plant was scored as a pollen shedding plant. The HMF was then calculated by dividing the number of pollen shedding plants by the total number of haploid plants per genotype:

$$\begin{array}{l} \text{HMF}=\;\frac{\text{number of pollen shedding plants}}{\text{total number of haploid plants}}\times 100\% \end{array}$$

The HMF was transformed to *arcsin* (HMF) to achieve normality of residuals. Best linear unbiased estimators (BLUE) for each haploid genotype (***G***_*i*_) treated as fixed effect were calculated with the PROC MIXED procedure of SAS software, both across the two genetic backgrounds as well as separately for each of them:

$$\begin{array}{l} \text{Across backgrounds}:y_{ijk}=\mu +G_{i}+B_{j}+{GB}_{ij}+E_{k}+\varepsilon_{ijk}\\ \text{“Zheng}58\text{” or }“\text{Mo}17\text{” background}:y_{ik}=\mu +G_{i}+E_{k}+\varepsilon_{ik} \end{array}$$

where *y*_*ijk*_ is the observed phenotype in the *k*^*th*^ environment for the haploid from the cross of the *i*^*th*^ genotype with the *j*^*th*^ background tester, μ is the grand mean, *G*_*i*_ is the effect of the *i*^*th*^ genotype, *B*_*j*_ is the effect of the *j*^*th*^ background tester, *GB*_*ij*_ is the interaction effect of the *i*^*th*^ genotype and *j*^*th*^ background tester, *E*_*k*_ is the random effect of *k*^*th*^ environment, ε_*ijk*_ is the error term confounded with the genotype-by-environment interaction, which follows an independent normal distribution $N\left(0,\sigma_{e}^{2}\right)$.

Broad sense heritability (*h*^2^) was calculated as follows:

$$\begin{array}{l} \text{Across backgrounds}:h^{2}=\frac{\sigma_{G}^{2}}{\sigma_{G}^{2}+\frac{\sigma_{G\times B}^{2}}{n_{T}}+\frac{\sigma_{e}^{2}}{n_{T}\times n_{E}}}\\ “\text{Zheng}58\text{” or }“\text{Mo}17\text{” background}:h^{2}=\frac{\sigma_{G}^{2}}{\sigma_{G}^{2}+\frac{\sigma_{e}^{2}}{n_{E}}} \end{array}$$

where $\sigma_{G}^{2}$ is the genotypic variance, $\sigma_{G\times B}^{2}$ is the genotype × background tester interaction variance, $\sigma_{e}^{2}$ is the residual error variance, *n*_*T*_ and *n*_*E*_ are the number of background testers and environments, respectively. All variance components were estimated by SAS using REML method with the PROC VARCOMP assuming random effects.

The Shannon-Weaver index (H') (Poole, 1974; Yang et al., 2010), measures the phenotypic diversity in categorical data. Briefly, the phenotypic values were subdivided into 10 classes by the means with an interval of 0.5 SD (Standard Deviation) of shedding rate, then number (*n*) and frequency (*p*_*i*_) was counted for each phenotypic class. The index was defined by Poole (1974) as

$$\begin{array}{c} H^{{}^{\prime }}=\overset{n}{\underset{i=1}{\sum }}p_{i}ln{\;p}_{i} \end{array}$$

### Genotyping and quality control

Genotypic data was obtained by SNP chip genotyping and RNA sequencing, as described in previous studies (Fu et al., 2013; Li et al., 2013; Yang et al., 2014). Briefly, the whole panel of 513 maize inbred lines was genotyped with the Maize SNP50 BeadChip (Illumina) containing 56,110 SNPs (Li et al., 2012). RNA sequencing was performed on immature seeds for 368 out of the 513 maize inbreds using 90-bp paired-end Illumina sequencing, resulting in 2445.9 GB of raw sequencing data. Five hundred fifty-eight thousand six hundred fifty high quality SNPs were obtained by combining results from the two genotyping platforms (Fu et al., 2013; Li et al., 2013). After KNN imputation based on identity-by-descent (IBD) for the remaining 145 lines, all 513 lines had 556,809 SNP marker types (Yang et al., 2014). The number of alleles, minor allelic frequencies (MAF), gene diversity, and polymorphic information content (PIC) were calculated using PowerMarker version 3.25 (Figures S2, S3) (Liu and Muse, 2005). Of the 556,809 SNPs, 425,597 SNPs had missing data <10% and a MAF >5% and were selected for the association analysis of the 481 lines in this study.

### Genome-wide association mapping

Population structure was estimated using the STRUCTURE program version 2.3 (Pritchard et al., 2000), which classified the 481 maize lines into four subgroups and yielded the population structure matrix *Q* (Li et al., 2012). Principal component analysis (PCA) was done based on 206,793 SNPs with a MAF ≥0.18 and a missing rate < 0.10 to obtain the *P* matrix with the *prcomp* function in R (Team, 2012). While *P* or *Q* can be used to capture major population stratification, kinship can be used to capture more subtle relationships. Consequently, 425,597 SNPs were used to estimate the relative kinship by TASSEL V5.0.6 (Bradbury et al., 2007) with the “pairwise IBS” option. To evaluate the resolution to be expected in association mapping, the linkage disequilibrium within the panel was evaluated by computing the parameter *r*^2^ between pairs of SNP markers in a sliding window of 50 markers using TASSEL V5.0.6 and tabulating the average *r*^2^ as a function of the physical distances between pairs of markers (Table S1, Figure S4).

For association mapping six models were compared, correcting for population structure (*Q, P*) and/or kinship (*K*): (1) the Naive model, without controlling for population structure and kinship; (2) the P model, only controlling for *P*; (3) the Q model, only controlling for *Q*; (4) the K model, only controlling for *K*; (5) the P+K model, controlling for both *P* and *K*; (6) the Q+K model, controlling for both *Q* and *K*. The Naive, P and Q model were performed using a general linear model (GLM) in TASSEL V5.0.6 (Yu et al., 2006; Bradbury et al., 2007; Zhang et al., 2009); the K, P+K, and Q+K models were performed using compressed mixed linear model (CMLM) in TASSEL. Quantile-quantile plots and association scan results showed the Q+K model to perform best and consequently, results are only shown for this model (Figures S5–S8). The genome-wide threshold for marker-trait associations was set at *P*-value < 0.10/(*N*/10) ($\hat{=}$ –log_10_ (*P*-value) > 5.63) in analogy to significance testing using the Bonferroni-Holm procedure (Holm, 1979), but taking the extremely high number of markers into account.

All candidate genes were annotated according to the information available in MaizeGDB database (http://www.maizegdb.org/gbrowse/maize_v2) and InterProScan (http://www.ebi.ac.uk/interpro/).

## Results

### Evaluation of haploid male fertility in maize

**Figure 1:**
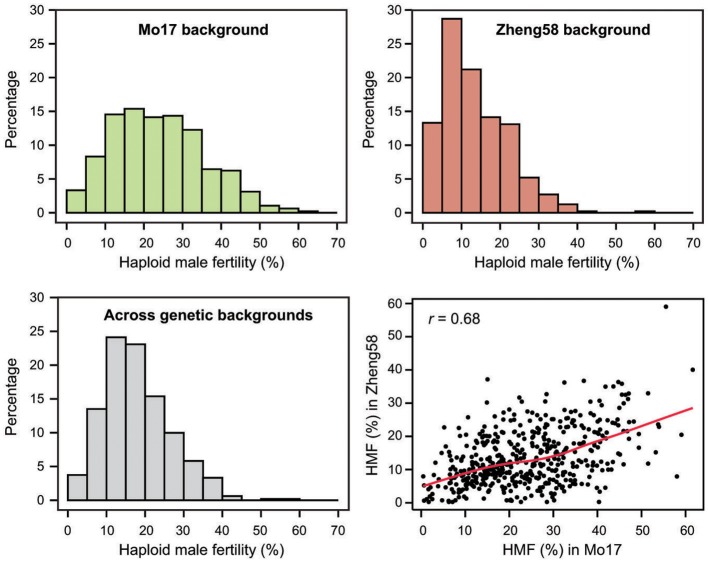
Histograms of the BLUEs for haploid male fertility (HMF) for each genetic background separately (“Mo17” and “Zheng58”) and across both genetic backgrounds, and correlation between the HMF BLUEs of the 481 genotypes in the two genetic backgrounds.

Haploid male fertility was assessed for 481 diverse maize lines crossed with “Mo17” and “Zheng58.” This revealed a large variation of the trait, ranging from zero to a maximum of 61.6% in the “Mo17” genetic background and 59.0% in the “Zheng58” background (Table 1, Figure 1). The three genotypes with the highest HMF in the “Mo17” background were “CIMBL61” (61.6%), “4F1” (59.1%), and “SY1035” (58.0%), and in the “Zheng58” background “RY684” (59.0%), “CIMBL61” (40.1%), and “B151” (37.2%). The mean HMF was 23.8% in the “Mo17” background and 13.5% in the “Zheng58” background. Of the two testers used here, “Mo17” had a higher HMF than “Zheng58,” and the HMF of “Mo17” of 32.4% was reduced to 15.1% in the “Zheng58” background. The correlation of HMF of the 481 maize lines in the two genetic backgrounds was 0.68 (*P* < 0.0001). The Shannon-Weaver index with 2.03 across the backgrounds and 2.05 in the “Mo17” background further confirmed the large phenotypic diversity present in this panel. The difference in HMF among all 481 maize lines was statistically highly significant (*P* < 0.0001), as was the interaction of genotype and genetic background (*P* < 0.01) as well as the difference between the two genetic backgrounds (*P* < 0.0001) and between the two environments (*P* < 0.0001) (Table S2). The estimated broad sense heritability was 0.65 across the two genetic backgrounds and 0.63 and 0.57 in the “Mo17” and “Zheng58” backgrounds, respectively.

**Table 1:** Summary statistics for haploid male fertility across both genetic backgrounds and separately for the “Mo17” and “Zheng58” background.

	**Across**	**Mo17**	**Zheng58**
Mean (%)	18.05	23.80	13.47
Min (%)	0.65	0.47	0.26
Max (%)	57.29	61.63	59.03
CV (%)	60.90	68.86	46.55
H'	2.03	2.05	1.99
$\sigma_{G}^{2}$	28.51	43.53	28.06
$\sigma_{G\times B}^{2}$	6.94		
$\sigma_{e}^{2}$	47.78	51.46	42.61
$h_{}^{2}$	0.65	0.63	0.57

*Mean and range, coefficient of variation (CV), Shannon-Weaver index (H′), genotypic variance ($\sigma_{G}^{2}$), genotype-by-background interaction variance ($\sigma_{G\times B}^{2}$), error variance ($\sigma_{e}^{2}$), and heritability (h^2^)*.

### Population structure and analysis of *qhmf4*

**Figure 2:**
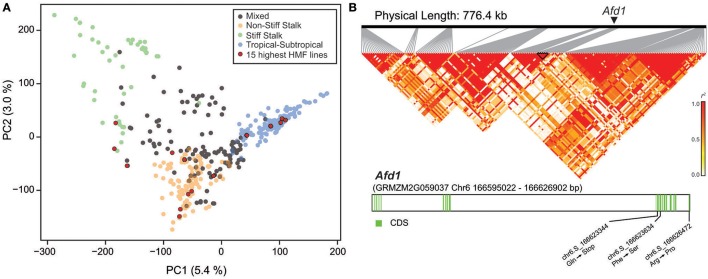
**(A)** Principal coordinate plot illustrating the population structure and highlighting the 15 genotypes with the highest haploid male fertility (HMF). **(B)** Linkage disequilibrium in the *qhmf4* genomic region, with the position of the *Afd1* gene indicated. Structure of the *Afd1* gene, with the positions of the three SNPs leading to amino acid changes.

The diversity panel comprises lines of different origin, i.e., Stiff Stalk, NSS, TST, and mixed origin (Figure 2A). The mean HMF of these subpopulations was 19.84% for Stiff Stalk, 19.83% for NSS, 16.39% for TST, and 18.62% for the mixed group. Interestingly, the 15 lines with the highest HMF were found to originate from all four different subpopulations. Next, we aimed to evaluate the previously identified *qhmf4* QTL in this diversity panel in more detail. We used the two markers identified by Ren et al. (2017) to flank the ~800 kb region encompassing *qhmf4* and analyzed the linkage disequilibrium (LD) among the 160 markers in this chromosomal region (Figure 2B). This revealed a complex LD pattern with several blocks of markers in higher LD but low LD between them. In addition, we investigated polymorphisms in *Absence of* first *division1* (*Afd1*), a potential candidate gene for *qhmf4* identified by Ren et al. (2017). Five SNP polymorphisms were found in the *Afd1* coding sequence, of which one resulted in a stop codon at position chr6:166623344 and two in an amino acid exchange (Figure 2B). However, none of these three polymorphisms was significantly associated with HMF in this panel.

### Association mapping of haploid male fertility in two genetic backgrounds

**Figure 3:**
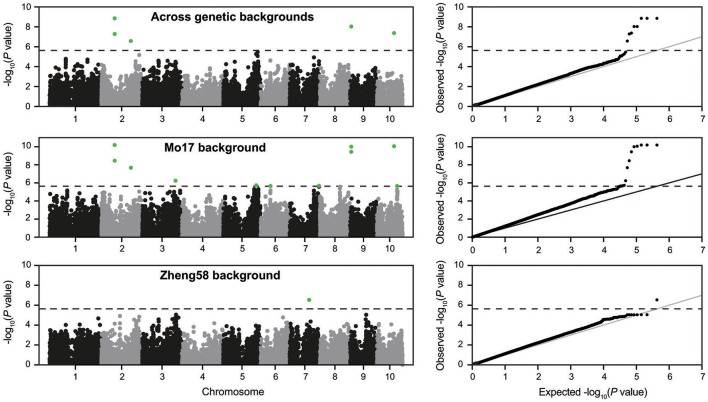
Manhattan plots from the association scans for haploid male fertility across genetic backgrounds and in the “Mo17” and “Zheng58” backgrounds. The dashed horizontal line indicates the significance threshold. In addition the quantile-quantile plots for expected and observed –log_10_(*P*-values) are shown.

**Figure 4:**
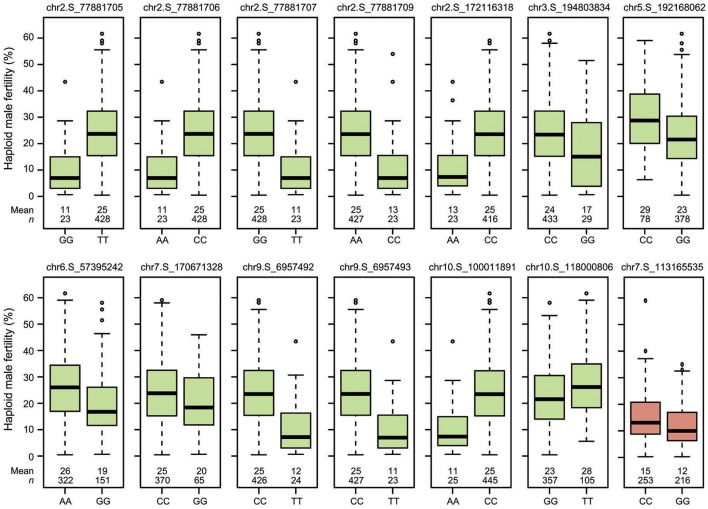
Boxplots showing the effects of the QTL identified in the “Mo17” (green) and “Zheng58” (red) genetic backgrounds on haploid male fertility.

Genome-wide association mapping yielded somewhat different results for the two genetic backgrounds (Figure 3). Significant association signals across genetic backgrounds and in the “Mo17” background were similar but different from the “Zheng58” background. Eight marker-trait associations were identified across both genetic backgrounds and 13 in the “Mo17” background. Only one SNP was significantly associated with HMF in the “Zheng58” background, however, with an alternative model (Q model) six marker-trait associations were found for “Zheng58” (Table S3). Thus, across all three association scans 14 SNPs were identified as significantly associated with haploid male fertility. The eight SNPs detected across backgrounds and in the “Mo17” background, were located in bins 2.05, 2.06, 9.01, and 10.04, with the number of SNPs per bin ranging from 1 to 4. Five SNPs were detected only in the “Mo17” background, that were located in bins 3.07, 5.05, 6.01, 7.05, and 10.04. The 14 significant SNPs jointly explained 22.5% of the total phenotypic variation across backgrounds, ranging from 0.01% to a maximum of 11.77% for the putative QTL identified on chromosome 2 (Table 2). The effects of these QTL ranged from 0.1 to 17.6% change in HMF and were expressed in Figure 4.

**Table 2:** Physical positions of 14 SNPs significantly associated with HFM based on QK model and the predicted function or homology of adjacent candidate genes.

**SNP**	**Chr**.	**Physical position^a^**	**Background^b^**	**Bin**	**Alleles^c^**	**MAF**	***p*-value**	***R*^2^^d^ for across (%)**	***R*^2^ for Mo17 (%)**	***R*^2^ for Zheng58 (%)**	**Candidate gene^e^**	**Annotation^f^**
chr2.S_77881705	2	77881705	Across, Mo17	2.05	T/*G*	0.051	1.38E-09	11.77	10.74	6.46	GRMZM2G174092	Unknown
chr2.S_77881706	2	77881706			C/*A*	0.051	1.38E-09					
chr2.S_77881707	2	77881707			G/*T*	0.051	1.38E-09					
chr2.S_77881709	2	77881709			A/*C*	0.050	3.62E-09	0.68	0.58	0.41		
chr2.S_172116318	2	172116318		2.06	C/*A*	0.052	2.13E-08	0.02	0.01	0.01	GRMZM2G474459	Unknown
chr3.S_194803834	3	194803834	Mo17	3.07	C/*G*	0.068	5.88E-07	1.17	1.14	0.59	GRMZM2G111657	Exocytosis
chr5.S_192168062	5	192168062		5.05	G/*C*	0.171	1.83E-06	2.75	2.26	1.72	GRMZM2G056236	Sexual reproduction
chr6.S_57395242	6	57395242		6.01	A/*G*	0.319	2.24E-06	3.30	5.71	0.34	GRMZM2G140867	Endopeptidase activity/threonine-type endopeptidase activity
chr7.S_113165535	7	113165535	Zheng58	7.02	C/*G*	0.462	7.63E-08	1.22	0.01	4.83	GRMZM2G029153	Transporter activity/transmembrane transporter activity/substrate-specific transmembrane transporter activity
chr7.S_170671328	7	170671328	Mo17	7.05	C/*G*	0.149	2.13E-06	0.28	1.02	0.04	GRMZM2G133275	Unknown
chr9.S_6957492	9	6957492	Across, Mo17	9.01	C/*T*	0.053	3.68E-10	0.01	0.05	0.01	GRMZM2G469593	Unknown
chr9.S_6957493	9	6957493			C/*T*	0.051	9.19E-09		0.16	0.20		
chr10.S_100011891	10	100011891		10.04	C/*A*	0.053	4.17E-08	0.21	0.58	0.00	GRMZM2G154667	RNA binding/translation initiation factor activity
chr10.S_118000806	10	118000806	Mo17	10.04	G/*T*	0.227	2.25E-06	1.12	1.03	0.58	GRMZM2G353213	Unknown

a Position in base pairs for the lead SNP according to version 2 of the B73 maize reference sequence (http://www.maizegdb.org/gbrowse/maize_v2).

b *Genetic background in which SNPs were significant*.

c *Major allele, minor allele; underlined bases are the minor alleles*.

d *Proportion of phenotypic variance explained by SNP*.

e *A plausible biological candidate gene in the locus or the nearest annotated gene to the lead SNP*.

f *Each candidate gene was annotated according to InterProScan (http://www.ebi.ac.uk/interpro/)*.

### Candidate genes of SNPs significantly associated with haploid male fertility

We next assessed the position of these SNPs on the maize genome, which revealed that two of them were adjacent to a gene (GRMZM2G469593) and the others were located within 9 genes (Table 2). Four significant SNPs were found in the gene GRMZM2G174092 located in bin 2.05, but at present the gene function is unknown. Interestingly, one of the genes (GRMZM2G056236) is annotated as being involved in sexual reproduction and may thus affect the restoration of haploid male fertility.

### Assessing the potential of genome-wide prediction

**Figure 5:**
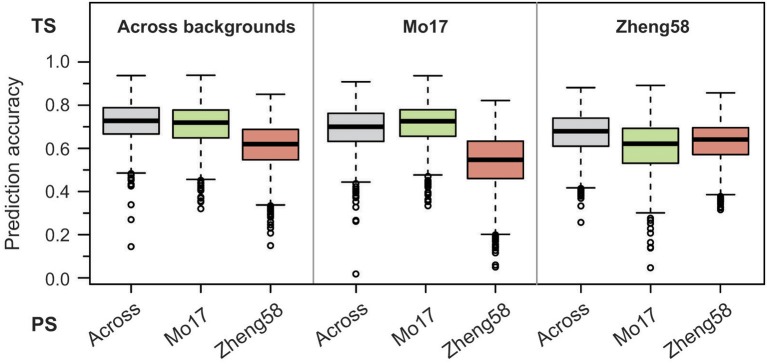
Genome-wide prediction for haploid male fertility. Prediction accuracy from fivefold cross-validation is shown for effect estimation in the training set (TS) comprising either the BLUEs across both genetic backgrounds, the “Mo17” or the “Zheng58” background, and subsequent prediction in the three sets of BLUEs as prediction set (PS).

Last, we employed a genome-wide prediction approach using either the BLUEs across genetic backgrounds, or the BLUEs from the “Mo17” or “Zheng58” background as training set for effect estimation. Prediction with fivefold cross-validation was then done for each training set in the same three sets of BLUEs. The medians of the obtained prediction accuracies ranged between 0.55 and 0.73 (Figure 5), and for the “Mo17” and “Zheng58” genetic backgrounds were higher if effect estimation was done in the same background. Across genetic backgrounds the cross-validated prediction accuracy (correlation *r* divided by the square root of the heritability) averaged 0.68, corresponding to a mean prediction ability (correlation between predicted and observed values) of 0.55.

## Discussion

### Large phenotypic variation of haploid male fertility in maize

For 481 lines from the diversity panel enough seeds were produced from the crosses with “Mo17” and “Zheng58” and the subsequent haploid induction. We chose this experimental design, as the DH production in applied maize breeding programs is also based on heterozygous plants and thus this setup is most realistic for practical maize breeding. In the field trials, we assessed whether or not a haploid plant produced pollen. Notably, however, there are varying degrees of pollen shedding and thus haploid male fertility. Ren et al. (2017) and Wu et al. (2017) evaluated anther emergence rate, anther emergence score, pollen production rate and pollen production score to assess HMF in a segregating population (Ren et al., 2017; Wu et al., 2017). However, as a single seed of a haploid plant provides already the desired DH line, we simplified phenotyping of pollen shedding and based on this calculated the HMF rate.

The heritability of HMF was moderately high, amounting to ~0.6 in both genetic backgrounds (Table 1). In combination with the observed trait distributions, this indicates a quantitative inheritance of this trait. Nevertheless, the effect of the genotype was highly significant (Table S2), illustrating the potential to improve HMF through breeding. Moreover, our results also revealed a significant difference between the two years (Table S2), illustrating that HMF is also affected by the environment. A more detailed knowledge of environmental factors leading to a high HMF might in the future allow to perform the DH production at specific environments that maximize haploid male fertility.

Interestingly, also the effect of the genetic background was significant and the mean HMF was 13.5% in the “Zheng58” background but 23.8% in the “Mo17” background (Table 1). Consequently, more lines with a high HMF rate could be identified in the “Mo17” background than in the “Zheng58” background. Notably, however, the genotype-by-genetic background effect was also significant and in line with this, we found the correlation between the HMF BLUEs in the “Mo17” and “Zheng58” backgrounds to be moderate with 0.68. This corroborates the conclusion of an at least in part additive genetic inheritance of haploid male fertility, but also indicates the contribution of epistatic effects. Consequently, lines with high trait values should be identified for an improvement of the trait through recurrent selection. As demonstrated here, such genotypes can indeed be identified by screening maize genetic diversity. HMF reached up to 61.6% in the “Mo17” background and in total 54 lines from the diversity panel exhibited a HMF rate >40%. Interestingly, these 54 lines do not appear to be related by origin, as they stem from all three genetic subgroups present in this panel. This further substantiates the conclusion of a complex genetic control underlying this trait and suggests that these lines may achieve their high HMF through different QTL or alleles thereof, which offers the potential to further increase HMF by pyramiding such QTL. Achieving this goal would, however, profit from a better understanding of the genetic architecture underlying HMF and potentially the identification of QTL to be used in marker-assisted selection.

### The genetic architecture of haploid male fertility

Our genome-wide scan revealed 14 marker-trait associations that were significant in either the “Mo17” or the “Zheng58” background. Jointly, these putative QTL explained only 22.5% of the phenotypic variance. This corroborates the conclusion of a quantitative nature of HMF and a complex genetic architecture, which was further substantiated by the genome-wide prediction approach. This approach allows to capture QTL with effects too small to be detected in association mapping. The predictive power of this approach was higher than that of the identified QTL, illustrating the contribution of additional small-effect QTL to haploid male fertility. The strongest QTL identified here was located on chromosome 2 and explained 11.77% of the phenotypic variance, and can thus be classified as a medium-effect QTL. Interestingly, only 5.1% of the lines carry the advantageous allele at this QTL, illustrating the potential of this locus for introgression in elite maize breeding programs that utilize DH production. A previous study based on a biparental population reported four QTL that were stable across three environments, *qhmf1, qhmf2, qhmf3*, and *qhmf4*, located in bins 1.11, 3.06/7, 4.02/03, and 6.07, respectively (Ren et al., 2017). While our putative QTL identified in bin 3.07 may correspond to *qhmf2*, the other association signals do not appear to correspond to the previously identified QTL. Thus, no QTL was found in our association study on chromosome 6 where *qhmf4* was recently identified as major QTL. This may be due to the lack of markers in sufficient linkage disequilibrium (LD) with this QTL, which however, appears unlikely given the high number of genome-wide markers employed here. Alternatively, this may indicate that the *qhmf4* allele is rare, which would have prevented its detection in an association mapping approach. It must be noted, that such rare alleles cannot be identified by association mapping in diversity panels, as they are below the applied minor allele frequency of 5% and in addition would lack statistical power to be detected. In combination with the results obtained here, this indicates that QTL alleles increasing HMF may generally be rare. Thus, while not identified here, major QTL for HMF may nevertheless be present in maize, but may be rare or even unique to certain lines. A consequent next step will therefore be the generation of biparental populations based on diverse lines with high HMF in order to investigate the genetic basis underlying their high trait values.

In the outcrossing crop maize in general, as well as in this particular diversity panel, LD decays comparably rapidly, potentially allowing to fine-map identified QTL (Figure S4). We therefore evaluated the annotation of the genes underlying the significant marker-trait associations (Table 2). Notably, this does not necessarily mean that these genes do indeed underlie the identified putative QTL. The cellular mechanism(s) resulting in spontaneous genome doubling of haploid cells is currently unknown, but may include endomitosis, endoreduplication, or somatic cell fusion (Jensen, 1974; Testillano et al., 2004; Vanous, 2011). *qhmf4* has recently been fine-mapped to a ~800 kb region that includes *Absence of* first *division1* (*Afd1*), a maize *rec8* homolog, as a potential candidate gene (Ren et al., 2017). In Arabidopsis, mutations in *rec8* together with mutations in two other genes lead to fertile haploid plants (Cifuentes et al., 2013). We identified three polymorphisms in *Afd1* that resulted in a premature stop codon or an amino acid exchange, however, none of them was significantly associated with HMF in this panel. While this does not rule out a role of *Afd1* in haploid male fertility, for example through rare polymorphisms not identified in this study, future research should also consider other candidate genes in the *qhmf4* region. In general, further work is required, particularly the cloning of the QTL, to better understand the biological pathways and regulatory mechanisms underlying HMF in maize and other species.

## Conclusions for maize DH breeding

In this study, we employed a large diversity panel to dissect the genetic architecture underlying HMF in maize. We observed a large variation for this important trait, with individual genotypes showing up to 60% haploid male fertility. These lines now represent an ideal starting point for a targeted introgression of high HMF into elite breeding material and a further improvement of the trait through recurrent selection. Genome-wide association mapping revealed only few putative QTL, thus substantiating the complex genetic nature of haploid male fertility. Nevertheless, considering the complexity and efforts required to phenotype haploid male fertility, marker-assisted selection based on validated QTL holds potential to assist breeding for this trait. If some underlying genes with larger effects can be identified, gene editing will become an attractive option to speed up their utilization in elite breeding material. Taken together, we identified substantial natural variation for HMF that can be exploited in maize breeding to make the generation of doubled haploid plants more efficient and thus economically attractive.

## Author contributions

SC managed the project. SC, HM, JY, WL, GL, YZ, DZ, XY, and JL designed and executed the experiment. GL and WL performed data analysis. GL, WL, and TW wrote the manuscript. All authors read and approved the final manuscript.

### Conflict of interest statement

The authors declare that the research was conducted in the absence of any commercial or financial relationships that could be construed as a potential conflict of interest.

## Supplementary material

Figure S1 Principal coordinate analysis plot for 481 maize inbred lines. Blue, green, red, and black color represent Stiff Stalk, Tropical-Subtropical, Non-Stiff Stalk, and the mixed subgroup, respectively.

Figure S2 The distribution of minor allele frequency for the 425,597 high quality SNPs.

Figure S3 Boxplots of summary statistics for 425,597 SNPs in all inbred lines from the diversity panel and in each subpopulation. **(A)** Minor allele frequency, **(B)** Gene diversity, **(C)** PIC.

Figure S4 Linkage disequilibrium (LD) across the 10 chromosomes in the maize diversity panel of 481 lines. The X-axis indicates the physical distance between SNPs within the same chromosome and the Y-axis indicates the LD (*r*^2^).

Figure S5 Quantile-quantile plots of estimated −log_10_(*P*-value) from association analysis for haploid male fertility using six methods, **(A)** across genetic backgrounds, and within the **(B)** “Mo17” and **(C)** “Zheng58” backgrounds. The gray solid line is the expected line under the null distribution where there are few true marker associations. The observed *P*-values are expected to nearly follow the expected *P*-values. Deviations from expectation demonstrate that the statistical analysis may cause spurious association. The significance level is marked by the horizontal dotted line.

Figure S6 Manhattan plots for haploid male fertility across genetic backgrounds assessed with six models. The dashed horizontal line indicates the genome-wide significance threshold.

Figure S7 Manhattan plots for haploid male fertility in the “Mo17” background assessed with six models. The dashed horizontal line indicates the genome-wide significance threshold.

Figure S8 Manhattan plots for haploid male fertility in the “Zheng58” background assessed with six models. The dashed horizontal line indicates the genome-wide significance threshold.

Table S1 Average linkage disequilibrium between marker pairs according to their physical distance, shown for all 10 chromosomes.

Table S2 ANOVA for the arcsine transformed haploid male fertility values.

Table S3 Physical positions of 6 SNPs significantly associated with HFM based on Q model under Zheng58 background and the predicted function or homology of adjacent candidate genes.
