# Supplementary figures and images for: Genome-Wide Association Study of Haploid Male Fertility in Maize (Zea Mays L.)
Source: Front Plant Sci. 2018 Jul 17;9:974. doi: 10.3389/fpls.2018.00974 (PMC6057118; doi:10.3389/fpls.2018.00974)

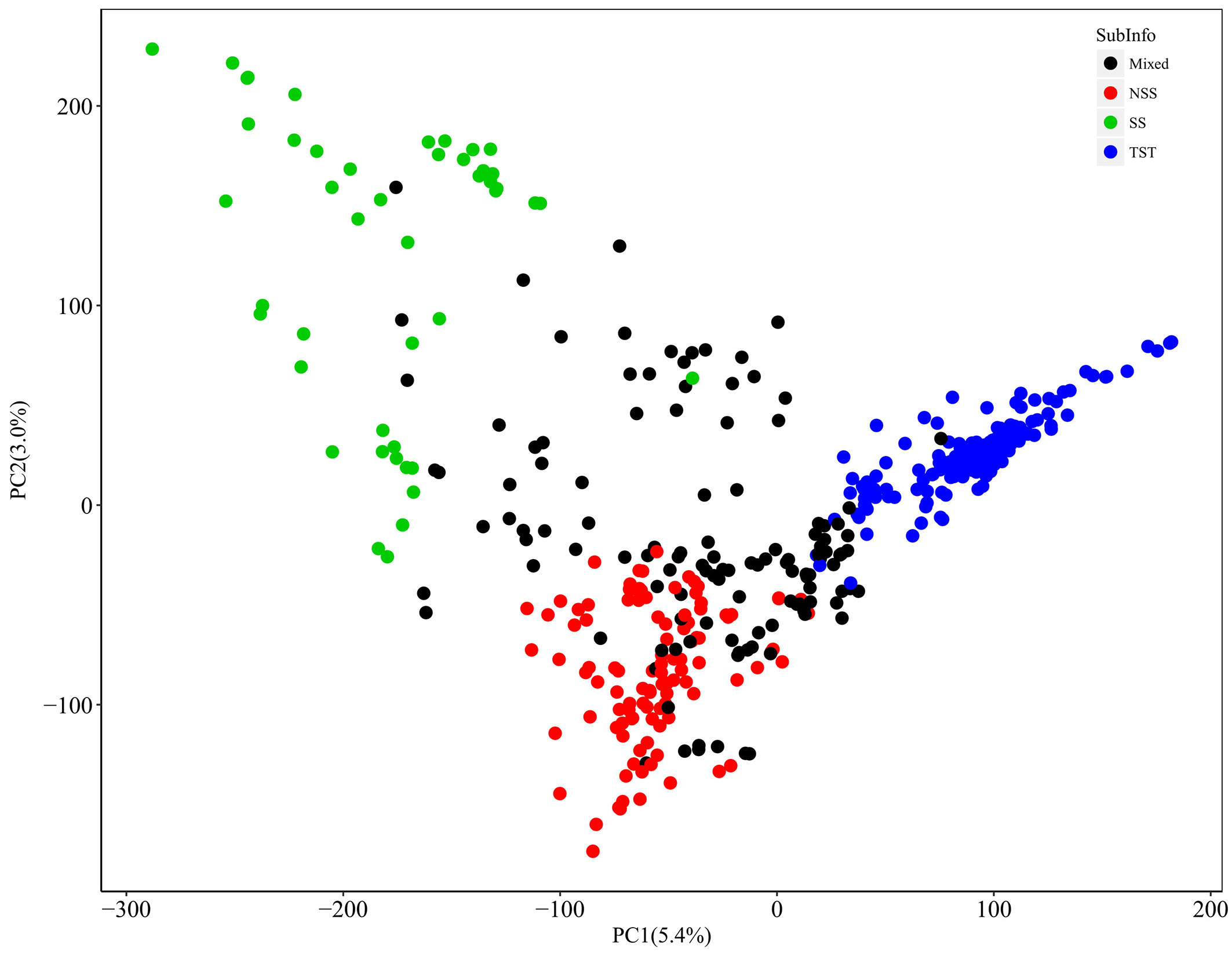

Supplement: Figure S1 — Principal coordinate analysis plot for 481 maize inbred lines. Blue, green, red, and black color represent Stiff Stalk, Tropical-Subtropical, Non-Stiff Stalk, and the mixed subgroup, respectively. [file Image_1.JPEG]

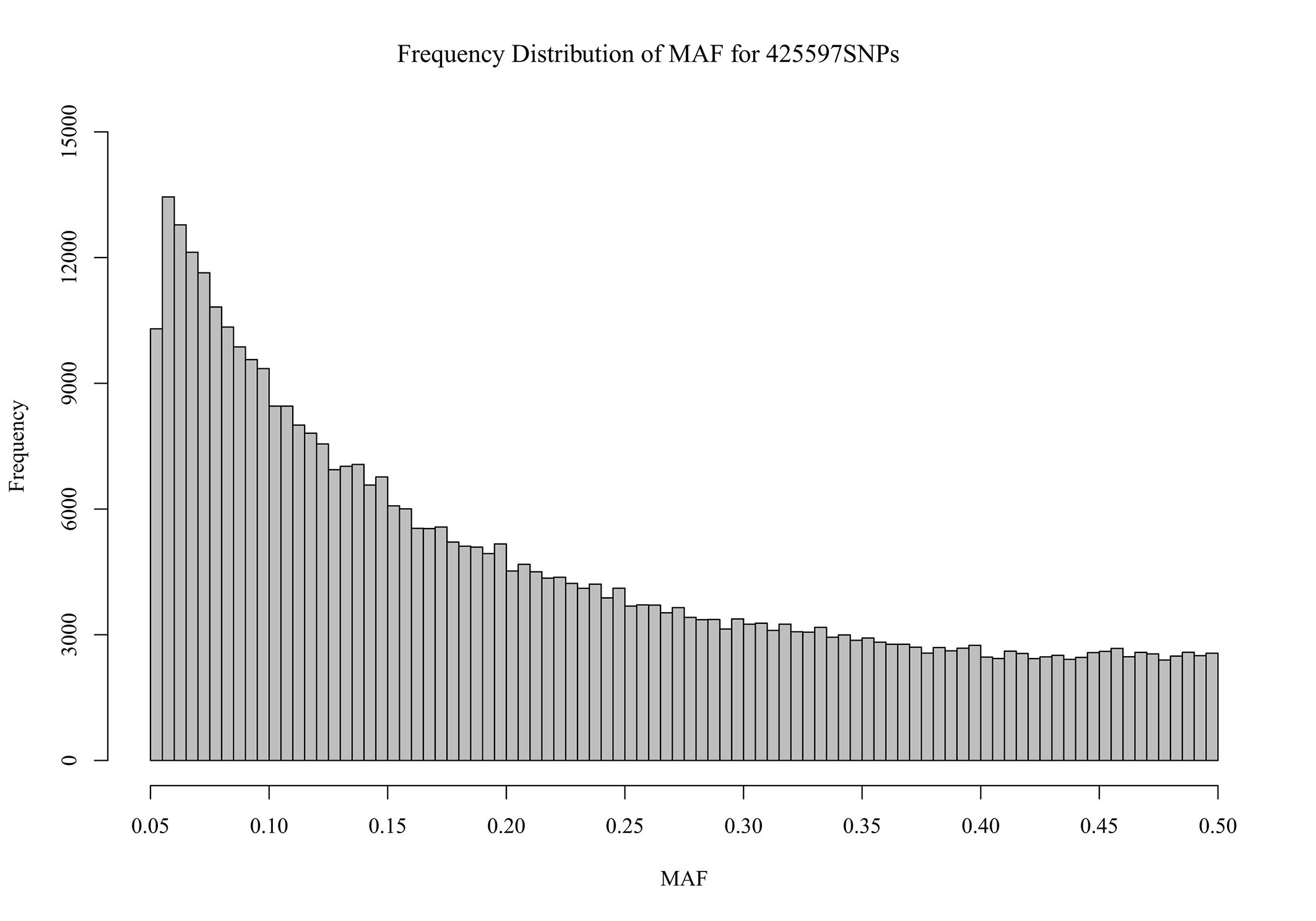

Supplement: Figure S2 — The distribution of minor allele frequency for the 425,597 high quality SNPs. [file Image_2.JPEG]

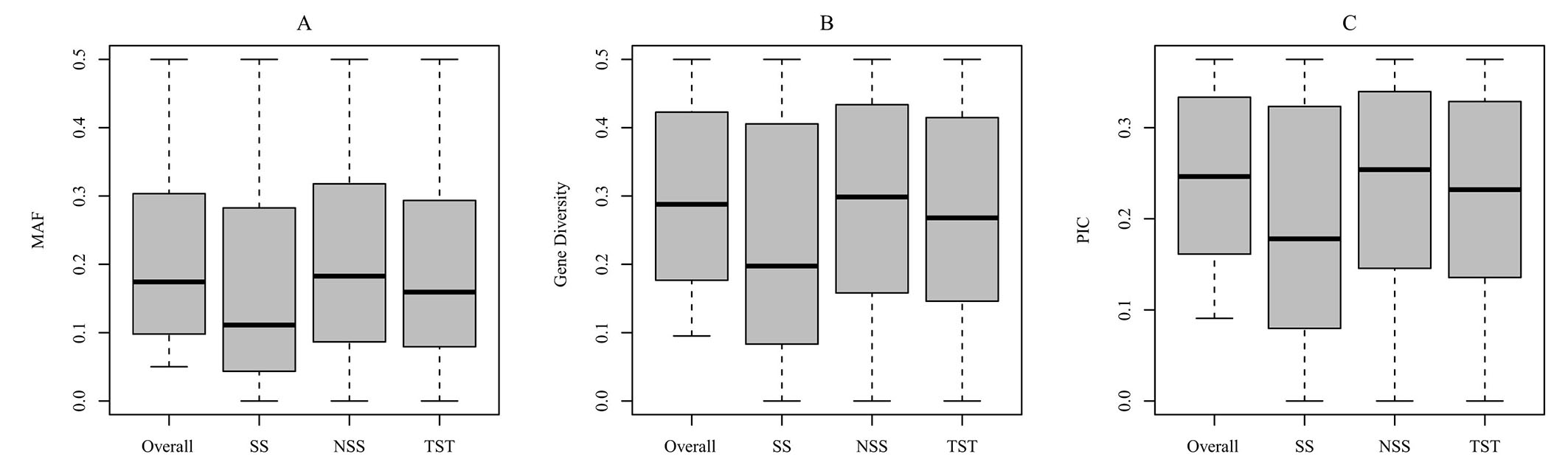

Supplement: Figure S3 — Boxplots of summary statistics for 425,597 SNPs in all inbred lines from the diversity panel and in each subpopulation. (A) Minor allele frequency, (B) Gene diversity, (C) PIC. [file Image_3.JPEG]

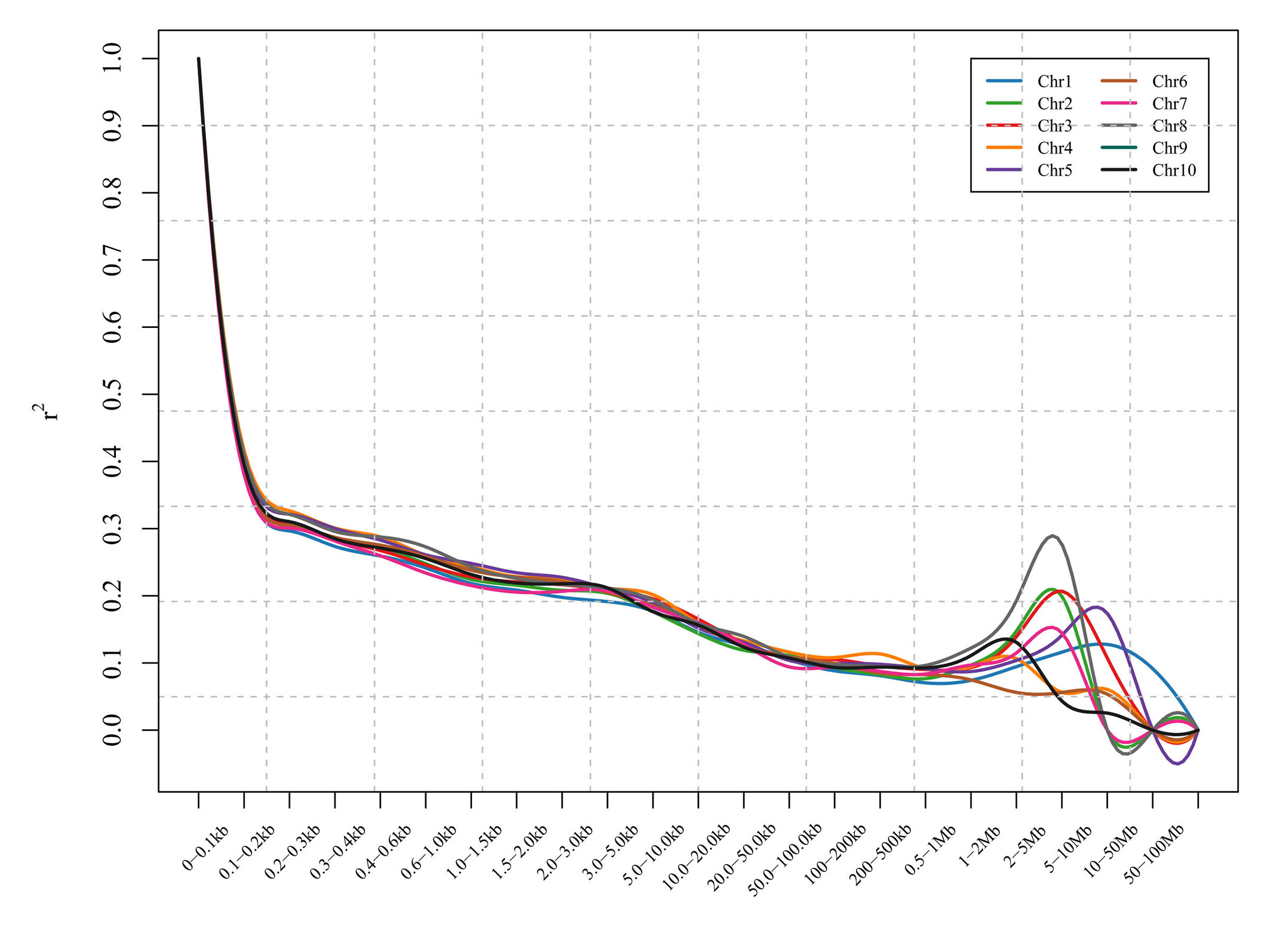

Supplement: Figure S4 — Linkage disequilibrium (LD) across the 10 chromosomes in the maize diversity panel of 481 lines. The X-axis indicates the physical distance between SNPs within the same chromosome and the Y-axis indicates the LD (r2). [file Image_4.JPEG]

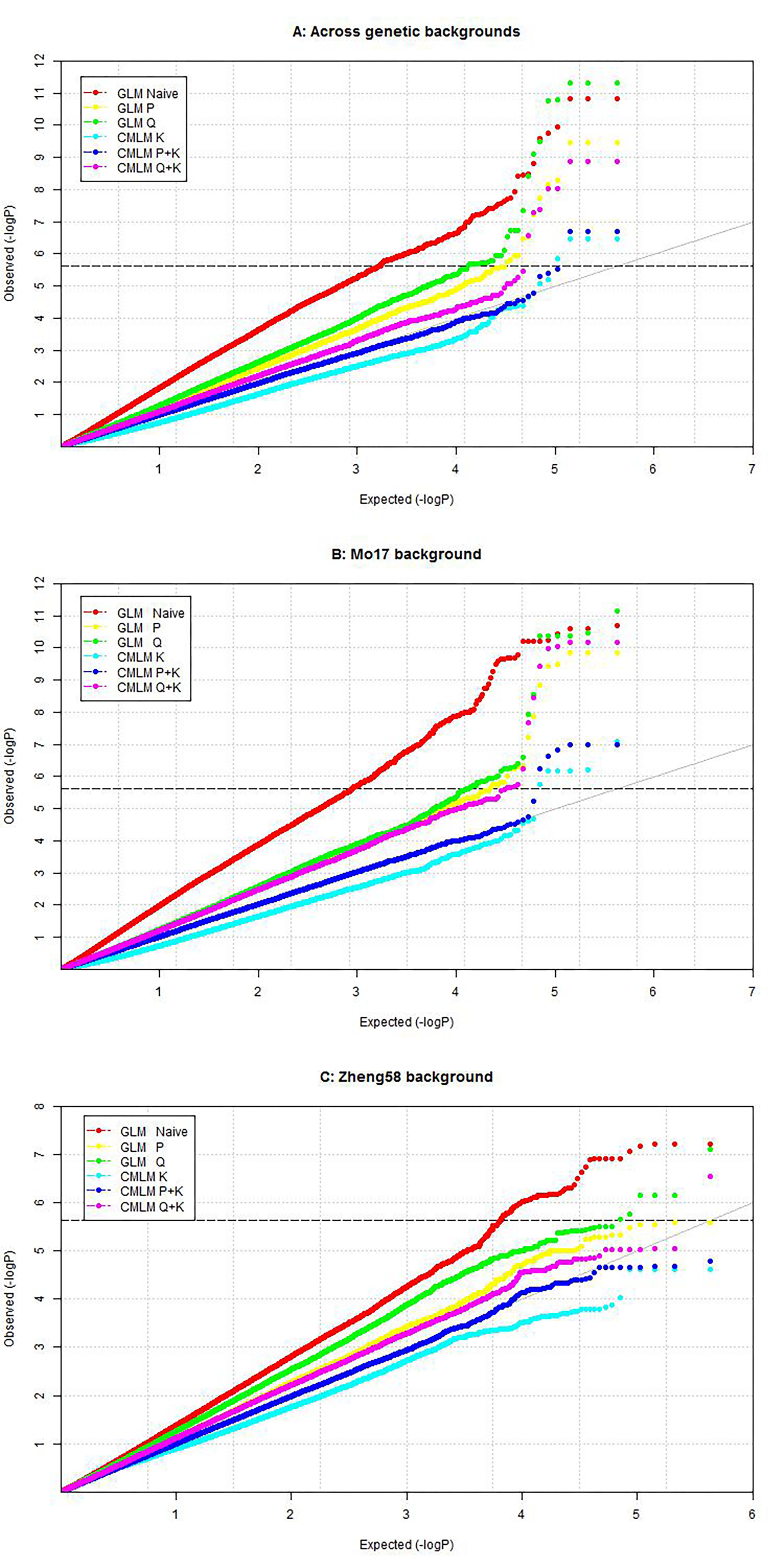

Supplement: Figure S5 — Quantile-quantile plots of estimated −log10(P-value) from association analysis for haploid male fertility using six methods, (A) across genetic backgrounds, and within the (B) “Mo17” and (C) “Zheng58” backgrounds. The gray solid line is the expected line under the null distribution where there are few true marker associations. The observed P-values are expected to nearly follow the expected P-values. Deviations from expectation demonstrate that the statistical analysis may cause spurious association. The significance level is marked by the horizontal dotted line. [file Image_5.JPEG]

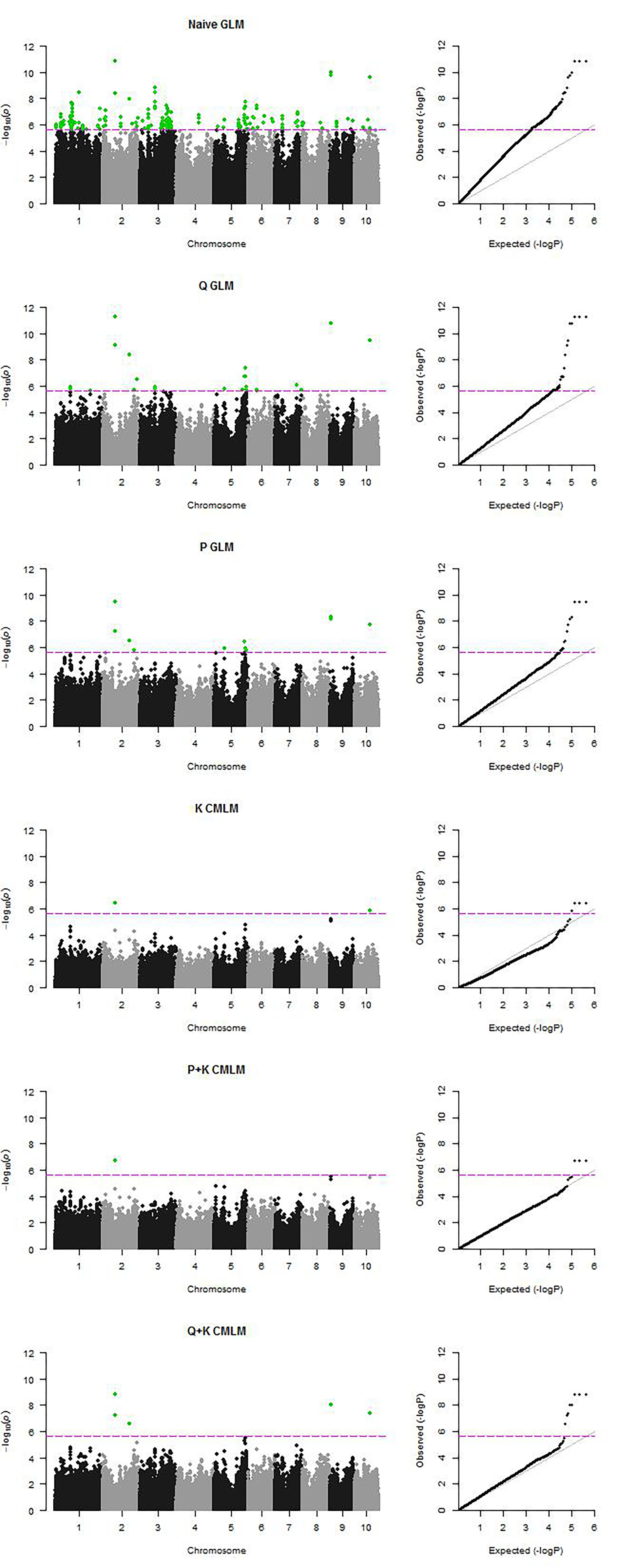

Supplement: Figure S6 — Manhattan plots for haploid male fertility across genetic backgrounds assessed with six models. The dashed horizontal line indicates the genome-wide significance threshold. [file Image_6.JPEG]

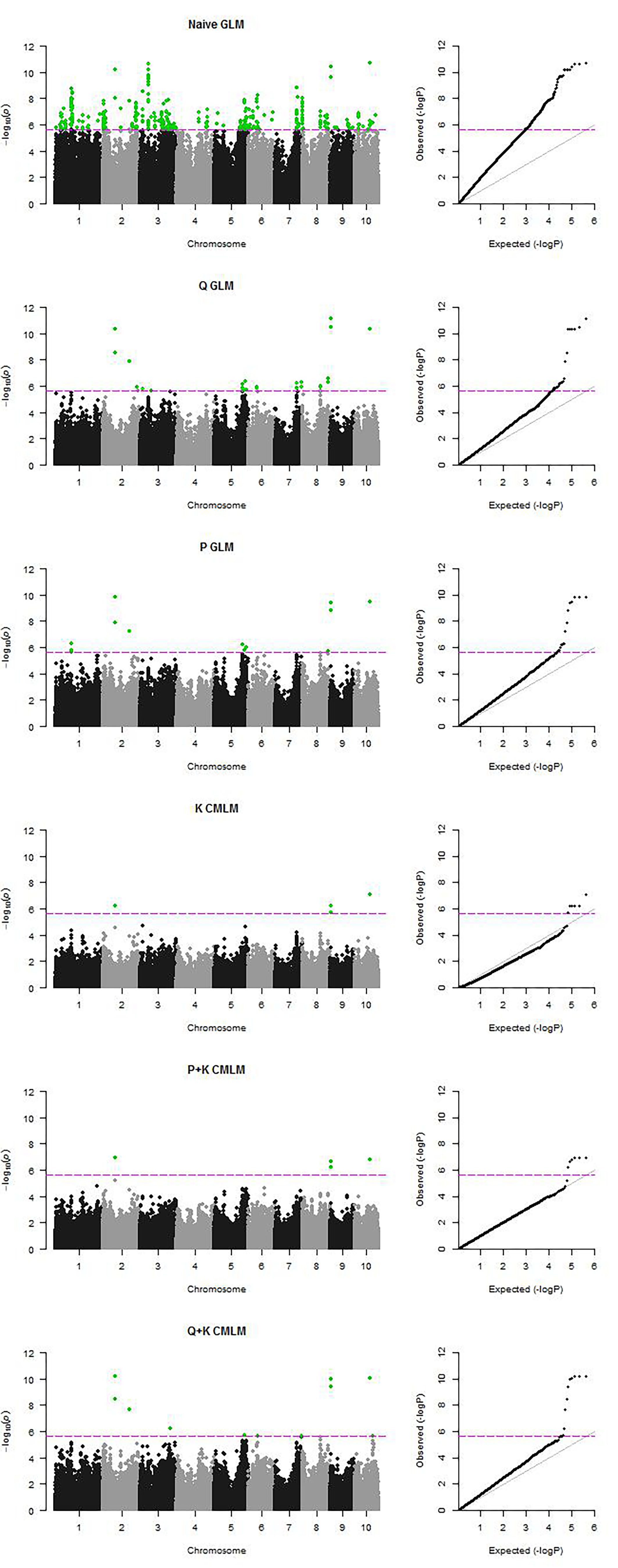

Supplement: Figure S7 — Manhattan plots for haploid male fertility in the “Mo17” background assessed with six models. The dashed horizontal line indicates the genome-wide significance threshold. [file Image_7.JPEG]

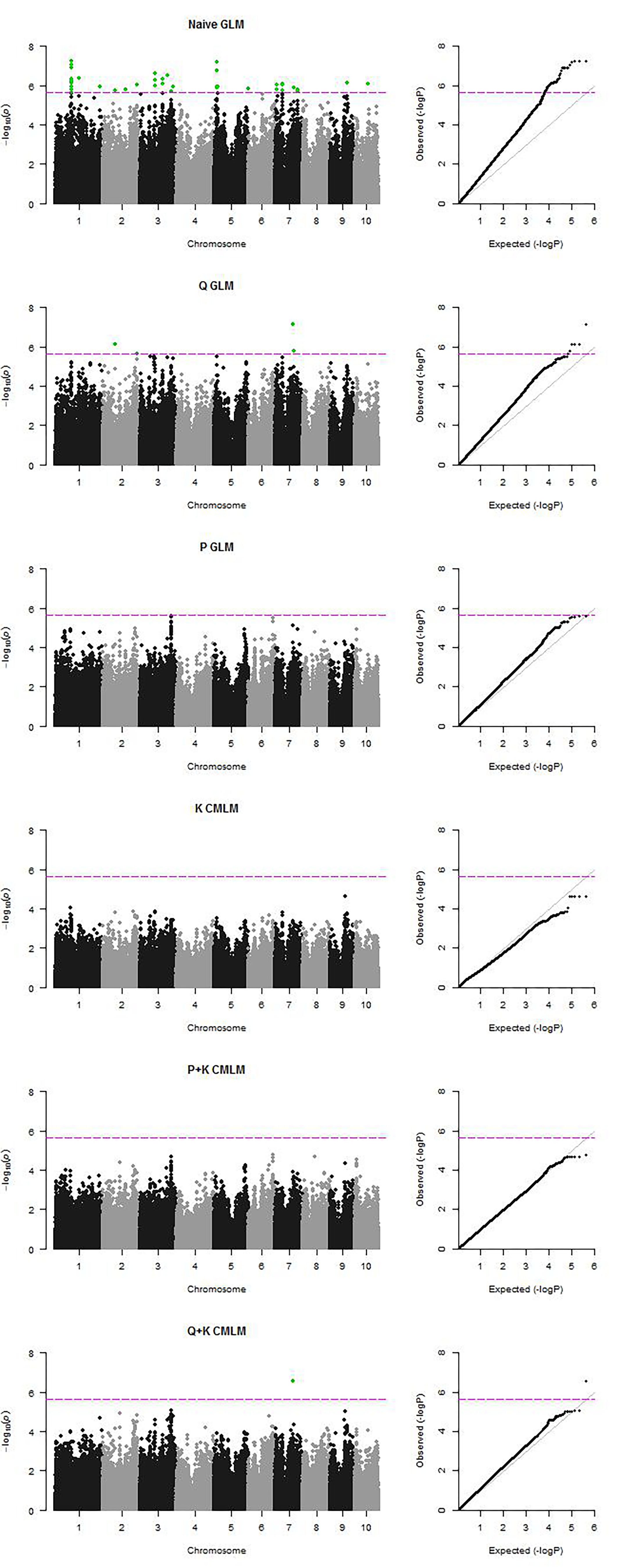

Supplement: Figure S8 — Manhattan plots for haploid male fertility in the “Zheng58” background assessed with six models. The dashed horizontal line indicates the genome-wide significance threshold. [file Image_8.JPEG]
