# Supplementary material for: Genome-Wide Association Study of Haploid Male Fertility in Maize (Zea Mays L.)
Source: Front Plant Sci. 2018 Jul 17;9:974. doi: 10.3389/fpls.2018.00974 (PMC6057118; doi:10.3389/fpls.2018.00974)
Supplement: Table S1 — Average linkage disequilibrium between marker pairs according to their physical distance, shown for all 10 chromosomes. [file Table_1.DOCX]

**Table S1** Average linkage disequilibrium between marker pairs according to their physical distance, shown for all ten chromosomes.

| Dist | Chr1 | Chr2 | Chr3 | Chr4 | Chr5 | Chr6 | Chr7 | Chr8 | Chr9 | Chr10 | Average | Stdev | CV |
| --- | --- | --- | --- | --- | --- | --- | --- | --- | --- | --- | --- | --- | --- |
| 0-0.1 kb | 0.38 | 0.39 | 0.39 | 0.42 | 0.40 | 0.39 | 0.38 | 0.41 | 0.40 | 0.39 | 0.40 | 0.01 | 0.03 |
| 0.1-0.2 kb | 0.30 | 0.31 | 0.31 | 0.33 | 0.32 | 0.31 | 0.30 | 0.32 | 0.31 | 0.31 | 0.31 | 0.01 | 0.03 |
| 0.2-0.3 kb | 0.27 | 0.28 | 0.28 | 0.30 | 0.30 | 0.29 | 0.28 | 0.30 | 0.29 | 0.29 | 0.29 | 0.01 | 0.03 |
| 0.3-0.4 kb | 0.26 | 0.27 | 0.27 | 0.29 | 0.28 | 0.28 | 0.26 | 0.29 | 0.27 | 0.28 | 0.27 | 0.01 | 0.04 |
| 0.4-0.6 kb | 0.24 | 0.25 | 0.25 | 0.26 | 0.26 | 0.26 | 0.23 | 0.27 | 0.26 | 0.26 | 0.25 | 0.01 | 0.04 |
| 0.6-1.0 kb | 0.22 | 0.22 | 0.23 | 0.24 | 0.25 | 0.24 | 0.22 | 0.24 | 0.23 | 0.24 | 0.23 | 0.01 | 0.05 |
| 1.0-1.5 kb | 0.21 | 0.22 | 0.22 | 0.23 | 0.23 | 0.23 | 0.21 | 0.23 | 0.22 | 0.22 | 0.22 | 0.01 | 0.04 |
| 1.5-2.0 kb | 0.20 | 0.21 | 0.22 | 0.23 | 0.23 | 0.22 | 0.21 | 0.22 | 0.22 | 0.21 | 0.21 | 0.01 | 0.04 |
| 2.0-3.0 kb | 0.19 | 0.20 | 0.21 | 0.21 | 0.21 | 0.20 | 0.21 | 0.21 | 0.21 | 0.21 | 0.21 | 0.01 | 0.03 |
| 3.0-5.0 kb | 0.18 | 0.18 | 0.20 | 0.20 | 0.19 | 0.19 | 0.18 | 0.19 | 0.18 | 0.20 | 0.19 | 0.01 | 0.05 |
| 5.0-10.0 kb | 0.15 | 0.14 | 0.17 | 0.16 | 0.15 | 0.16 | 0.16 | 0.16 | 0.16 | 0.16 | 0.16 | 0.01 | 0.04 |
| 10.0-20.0 kb | 0.13 | 0.12 | 0.13 | 0.13 | 0.13 | 0.12 | 0.13 | 0.14 | 0.12 | 0.12 | 0.13 | 0.01 | 0.05 |
| 20.0-50.0 kb | 0.10 | 0.11 | 0.11 | 0.12 | 0.10 | 0.11 | 0.09 | 0.11 | 0.11 | 0.13 | 0.11 | 0.01 | 0.07 |
| 50.0-100.0 kb | 0.09 | 0.09 | 0.11 | 0.11 | 0.10 | 0.10 | 0.09 | 0.10 | 0.09 | 0.10 | 0.10 | 0.01 | 0.06 |
| 100-200 kb | 0.08 | 0.08 | 0.10 | 0.11 | 0.10 | 0.08 | 0.09 | 0.09 | 0.09 | 0.11 | 0.09 | 0.01 | 0.12 |
| 200-500 kb | 0.07 | 0.08 | 0.09 | 0.09 | 0.09 | 0.08 | 0.08 | 0.10 | 0.09 | 0.10 | 0.09 | 0.01 | 0.10 |
| 0.5-1 Mb | 0.07 | 0.10 | 0.09 | 0.09 | 0.09 | 0.07 | 0.10 | 0.12 | 0.11 | 0.07 | 0.09 | 0.02 | 0.17 |
| 1-2 Mb | 0.09 | 0.15 | 0.14 | 0.11 | 0.10 | 0.06 | 0.11 | 0.19 | 0.13 | 0.10 | 0.12 | 0.04 | 0.31 |
| 2-5 Mb | 0.12 | 0.20 | 0.21 | 0.06 | 0.14 | 0.06 | 0.14 | 0.28 | 0.04 | 0.13 | 0.14 | 0.08 | 0.55 |
| 5-10 Mb | 0.13 | 0.00 | 0.11 | 0.06 | 0.17 | 0.05 | 0.00 | 0.00 | 0.03 | 0.00 | 0.05 | 0.06 | 1.14 |
| 10-50 Mb | 0.09 | 0.00 | 0.00 | 0.00 | 0.00 | 0.00 | 0.00 | 0.00 | 0.00 | 0.00 | 0.01 | 0.03 | 3.16 |
| 50-100 Mb | 0.00 | 0.00 | 0.00 | 0.00 | 0.00 | 0.00 | 0.00 | 0.00 | 0.00 | 0.00 | 0.00 | 0.00 | - |

Kb = kilo base; StdDev = standard deviation; CV = coefficient of variation
