# Supplementary material for: Genome-Wide Association Study of Haploid Male Fertility in Maize (Zea Mays L.)
Source: Front Plant Sci. 2018 Jul 17;9:974. doi: 10.3389/fpls.2018.00974 (PMC6057118; doi:10.3389/fpls.2018.00974)
Supplement: Table S2 — ANOVA for the arcsine transformed haploid male fertility values. [file Table_2.DOCX]

**Table S2** ANOVA for the arcsine transformed haploid male fertility values.

| Source | DF | SS | MS | F value | P |
| --- | --- | --- | --- | --- | --- |
| Year | 1 | 19609.02 | 19609.02 | 412.76** | <0.0001 |
| Genotype | 480 | 77751.32 | 161.98 | 3.41** | <0.0001 |
| Background | 1 | 25987.77 | 25987.77 | 547.03** | <0.0001 |
| Genotype×Background | 480 | 29048.70 | 60.52 | 1.27** | 0.0013 |
| Error | 821 | 39003.22 | 47.51 |  |  |
| Total | 1783 | 198151.90 |  |  |  |

** indicates significance at *P* ≦ 0.01
