# Supplementary material for: Genome-Wide Association Study of Haploid Male Fertility in Maize (Zea Mays L.)
Source: Front Plant Sci. 2018 Jul 17;9:974. doi: 10.3389/fpls.2018.00974 (PMC6057118; doi:10.3389/fpls.2018.00974)
Supplement: Table S3 — Physical positions of 6 SNPs significantly associated with HFM based on Q model under Zheng58 background and the predicted function or homology of adjacent candidate genes. [file Table_3.DOCX]

**Table S3** Physical positions of 6 SNPs significantly associated with HFM based on Q model under Zheng58 background and the predicted function or homology of adjacent candidate genes.

| SNP | Chr. | Physical Position ^a^ | bin | Alleles ^b^ | MAF | p_value | R^2 c^ | Candidate Gene ^d^ | Annotation ^e^ |
| --- | --- | --- | --- | --- | --- | --- | --- | --- | --- |
| chr2.S_77881705 | 2 | 77881705 | 2.05 | T/G | 0.051 | 7.12E-07 | 5.38% | GRMZM2G174092 | Unknown |
| chr2.S_77881706 | 2 | 77881706 |  | C/A | 0.051 | 7.12E-07 |  |  |  |
| chr2.S_77881707 | 2 | 77881707 |  | G/T | 0.051 | 7.12E-07 |  |  |  |
| chr2.S_217119711 | 2 | 217119711 | 2.08 | G/A | 0.172 | 2.20E-06 | 3.74% | GRMZM2G031125 | iron ion binding /oxidoreductase activity/heme binding |
| chr7.S_113165535 | 7 | 113165535 | 7.02 | C/G | 0.462 | 7.63E-08 | 2.95% | GRMZM2G029153 | transporter activity/transmembrane transporter activity/substrate-specific transmembrane transporter activity |
| PZE-107064549 | 7 | 121404852 | 7.02 | G/A | 0.233 | 1.69E-06 | 0.99% | GRMZM2G318319 | Unknown |

**^a^ Position in base pairs for the lead SNP according to version 2 of the B73 maize reference sequence (**[**http://www.maizegdb.org/gbrowse/maize_v2**](http://www.maizegdb.org/gbrowse/maize_v2)**).**

**^b^ Major allele, minor allele; underlined bases are the minor alleles.**

**^c^ Proportion of phenotypic variance explained by SNP.**

**^d^ A plausible biological candidate gene in the locus or the nearest annotated gene to the lead SNP.**

**^e^ Each candidate gene was annotated according to InterProScan (**[**http://www.ebi.ac.uk/interpro/**](http://www.ebi.ac.uk/interpro/)**).**
